# Parallel Batch Scheduling of Deteriorating Jobs with Release Dates and Rejection

**DOI:** 10.1155/2014/270942

**Published:** 2014-07-21

**Authors:** Juan Zou, Cuixia Miao

**Affiliations:** ^1^School of Mathematical Sciences, Qufu Normal University, Qufu, Shandong 273165, China; ^2^School of Management Sciences, Qufu Normal University, Rizhao, Shandong 276826, China

## Abstract

We consider the unbounded parallel batch scheduling with deterioration, release dates, and rejection. Each job is either accepted and processed on a single batching machine, or rejected by paying penalties. The processing time of a job is a simple linear increasing function of its starting time. The objective is to minimize the sum of the makespan of the accepted jobs and the total penalty of the rejected jobs. First, we show that the problem is NP-hard in the ordinary sense. Then, we present two pseudopolynomial time algorithms and a fully polynomial-time approximation scheme to solve this problem. Furthermore, we provide an optimal *O*(*n*log⁡*n*) time algorithm for the case where jobs have identical release dates.

## 1. Introduction

The parallel batch scheduling problem has been extensively studied in the literature over the last decade. The fundamental model of the parallel batch scheduling problem was first introduced by Lee et al. [1] with the restriction that the number of jobs in each batch is bounded by a number *b*. This bounded model is motivated by the burn-in operations in semiconductor manufacturing. Brucker et al. [2] provided an extensive discussion of the unbounded version of the parallel batch scheduling problem. This unbounded model can be applied, for example, to situations where the batch contents are heated in a sufficiently large kiln. Cheng et al. [3], Deng et al. [4, 5], and Liu et al. [6, 7] presented new complexity results and approximation algorithms for the parallel batch scheduling problem subject to release dates. In addition, more recent developments of this topic can be found in [8].

In classical scheduling problems, it is assumed that the processing times of jobs are fixed parameters. In practice, however, we often encounter situations in which processing time increases over time. Examples can be found in financial management, steel production, firefighting, and so forth, where any delay in processing a job may increase its completion time. Such problems are generally known as scheduling with deterioration effects. Browne and Yechiali [9] first considered scheduling problem with linear deterioration. They provided the optimal solution for the single machine when the objective is to minimize makespan. In addition, they solved a special case when the objective function is to minimize the total weighted completion time. Mosheiov [10] considered single machine scheduling problems with simple linear deteriorating jobs. He showed that most commonly scheduling objectives, such as the makespan, the total flow time, the sum of weighted completion times, the maximum lateness, the total lateness, the maximum tardiness, and the number of tardy jobs, remain solvable in polynomial time. Alidaee and Womer [11] presented an extensive survey of different deteriorating models and scheduling problems. Cheng et al. [12] provided an updated survey of scheduling problems with time-dependent processing times. Li et al. [13] considered the problem of scheduling of deteriorating jobs with release dates on a single batching machine. Miao et al. [14] studied the scheduling problems with deteriorating jobs and release dates on a single (batching) machine to minimize the maximum lateness. Zou et al. [15] considered several uniform parallel machine scheduling problems with linear deteriorating jobs.

On the other hand, it is always assumed in traditional scheduling research that all the jobs have to be processed on some machines. In real applications, however, things may be more complicated. For example, due to limited resources, the scheduler may reject some jobs and thereby incur job-dependent penalties for each rejected job. Bartal et al. [16] first introduced the notion of rejection. They considered both the offline and online versions of scheduling problem and studied the multiprocessor scheduling problem to minimize the sum of the makespan of the accepted jobs and the total rejection penalty of the rejected jobs. Subsequently, the machine scheduling problem with rejection has received increasing research attention. Hoogeveen et al. [17] considered the offline multiprocessor scheduling problem with rejection where preemption is allowed. They provided a pseudopolynomial time algorithm for each problem. Lu et al. [18] considered the unbounded parallel batch machine scheduling problem with release dates and rejection. Engels et al. [19] studied the single machine scheduling with rejection to minimize the sum of the weighted completion times of the scheduled jobs and the total penalty of the rejected jobs. Shabtay et al. [20] recently presented an updated survey on offline scheduling with rejection. In addition, there has been some work on scheduling deteriorating jobs with rejection. For example, Cheng and Sun [21] considered the single machine scheduling problems with linear deteriorating jobs and rejection. Li and Yuan [22] studied several parallel machine scheduling problems in which the processing time of a job is a linear increasing function of its starting time and jobs can be rejected by paying penalties. To the best of our knowledge, no work has been done on the problem of scheduling deteriorating jobs with rejection on a single batching machine. Literature [20] on scheduling with rejection focuses on the following four models:

 
*P*1: to minimize the total integrated cost *F*_1_ + *F*_2_;
 
*P*2: to minimize *F*_1_ subject to $F_{2}\leq \bar{E}$;
 
*P*3: to minimize *F*_2_ subject to *F*_1_ ≤ *K*;
 
*P*4: to identify a Pareto-optimal solution for each Pareto-optimal point,

where *F*_1_ is the original scheduling objective function and *F*_2_ is total rejection cost. We discuss the first model with deterioration and release dates.

In this paper, we study parallel batch scheduling of deteriorating jobs with release dates and rejection. The paper is organized as follows. In Section 2, we formulate the problem under consideration and introduce some notations. In Section 3, we first show that the problem is binary NP-hard and provide two pseudopolynomial time algorithms. We also propose a fully polynomial-time approximation scheme for the problem and discuss one special case. In Section 4, we conclude the paper and suggest some topics for future research.

## 2. Problem Description and Notation

The problem considered in this paper can be formally described as follows. There are an independent job set *J* = {*J*_1_,…, *J*_*n*_} and a single batching machine. The machine can process up to *b* job simultaneously as a batch, where *b* ≥ *n*. The jobs processed in the same batch have the same starting time and completion time. The processing time of a batch is equal to the largest processing time of the jobs in the batch. Each job *J*_*j*_  (*j* = 1,…, *n*) has a release date *r*_*j*_, a deteriorating rate *b*_*j*_ > 0, and a rejection penalty *e*_*j*_. Each job *J*_*j*_  (*j* = 1,…, *n*) is either rejected with a rejection penalty *e*_*j*_ having to be paid or accepted and processed on the parallel batch machine. Denote by *R* and $\bar{R}=J\setminus R$ the set of rejected jobs and the set of accepted jobs, respectively. The actual processing time of job *J*_*j*_ is given by *p*_*j*_ = *b*_*j*_ *t*, where *t* ≥ *r*_*j*_ is the starting time of *J*_*j*_. We assume min⁡{*r*_*j*_∣*j* = 1,…, *n*} ≥ *t*_0_ > 0. For a given batch *B*, we denote its deteriorating rate and release date by *b*(*B*) and *r*(*B*), respectively. Then, *b*(*B*) = max⁡{*b*_*j*_ : *J*_*j*_ ∈ *B*} and *r*(*B*) = max⁡{*r*_*j*_ : *J*_*j*_ ∈ *B*}. Denote by *C*_*j*_ the completion time of the accepted job *J*_*j*_. We study the problem of minimizing the sum of the makespan of the accepted jobs (i.e., $C_{\max}(\bar{R})=\max\{C_{j}:J_{j}\in \bar{R}\}$) and the total penalty of the rejected jobs. Using the three-field notation of Graham et al. [23], we denote the problem by $1\left|p\text{-batch},r_{j},p_{j}=b_{j}t,rej,b\geq n\right|C_{\max}(\bar{R})+\sum_{J_{j}\in R}e_{j}$.

## 3. Main Results

### 3.1. NP-Hardness Proof

We first give a lemma, which will be used in what follows.

**Lemma 1 (see [10]).**

For the problem 1|*r*_*j*_ = *t*_0_, *p*_*j*_ = *b*_*j*_ *t*|*C*_max⁡_, the maximum completion time of the jobs is equal to *C*_max⁡_ = *t*_0_∏_*J*_*j*_∈*J*_(1 + *b*_*j*_) and it does not depend on the processing order of the jobs.

**Theorem 2 .**

The problem $1\left|p\text{-}batch,r_{j},p_{j}=b_{j}t,rej,b\geq n\right|C_{\max}(\bar{R})+\sum_{J_{j}\in R}e_{j}$ is binary NP-hard.

**Proof**

The decision version of the scheduling problem is clearly in NP. We use a reduction from the Equal Products Problem, which is NP-hard (see [24]).

An instance I of the Equal Products Problem is formulated as follows. Given a set *Q* = {1,2,…, *m*} and positive integers *a*_*i*_ for each *i* ∈ *Q* such that ∏_*i*∈*Q*_ *a*_*i*_ = *A* ^2^ for a certain positive integer *A*, does there exist a subset *S* ⊂ *Q* such that ∏_*i*∈*S*_ *a*_*i*_ = *A*?

For any given instance I of the Equal Products Problem, we construct a corresponding instance II of our problem as follows.

- There are *n* = 2*m* + 1 jobs.
- For *i* = 1, there exist job *J*_1_ with (*r*_1_, *b*_1_, *e*_1_) = (1, *A* ^2*m*^ − 1,2*A* ^*m*^2^+*m*+1^ + *A*) and job *J*_2_ with (*r*_2_, *b*_2_, *e*_2_) = (1, *A* ^2*m*^ *a*_1_ − 1, ln⁡*a*_1_).
- For each 2 ≤ *i* ≤ *m*, there exist job *J*_2*i*−1_ with (*r*_2*i*−1_, *b*_2*i*−1_, *e*_2*i*−1_) = (*A* ^∑_*k*=0_^*i*−2^(2*m*−2*k*)^, *A* ^2*m*−2(*i*−1)^ − 1,2*A* ^*m*^2^+*m*+1^ + *A*) and job *J*_2*i*_ with (*r*_2*i*_, *b*_2*i*_, *e*_2*i*_) = (*A* ^∑_*k*=0_^*i*−2^(2*m*−2*k*)^, *A* ^2*m*−2(*i*−1)^ *a*_*i*_ − 1, ln⁡*a*_*i*_).
- For *i* = 2*m* + 1, there exists only a job *J*_2*m*+1_ with (*r*_2*m*+1_, *b*_2*m*+1_, *e*_2*m*+1_) = (*A* ^*m*^2^+*m*+1^, 1,2*A* ^*m*^2^+*m*+1^ + *A*).
- The threshold value is given by *Y* = 2*A* ^*m*^2^+*m*+1^ + ln⁡*A*.

The decision asks whether there is a schedule such that $C_{\max}(\bar{R})+\sum_{J_{j}\in R}e_{j}\leq Y$.

It is clear that the reduction can be done in polynomial time, and it is easy to verify that *b*_*i*_ > 0 for *i* = 1,…, *m* + 1. Assume first that the Equal Products Problem has a solution *S* such that ∏_*i*∈*S*_ *a*_*i*_ = *A*. Then, we construct a schedule in the following way. If *i* ∈ *S*, we assign the jobs *J*_2*i*−1_ and *J*_2*i*_ as a batch *B*_*i*_. If *i* ∉ *S*, we assign the job *J*_2*i*−1_ as a batch *B*_*i*_. We assign the job *J*_2*m*+1_ as the last batch *B*_*m*+1_. The jobs are processed on the batch machine in the order of *B*_1_,…, *B*_*m*+1_. All other jobs are rejected. It is easy to verify that

(1) Cmax⁡(R¯)+∑Jj∈Rej =max⁡⁡{Am2+m∏i∈Sai,rm+1}(1+bm+1)+∑i∉Sln⁡ai =2Am2+m+1+ln⁡A=Y.

Conversely, suppose that there is a schedule *π* with $C_{\max}(\bar{R})+\sum_{J_{j}\in R}e_{j}\leq Y$. We need to show that there is a subset *S* for the Equal Products Problem with ∏_*i*∈*S*_ *a*_*i*_ = *A*. We have the following claims.

**Claim 1 .**

All jobs *J*_2*i*−1_ for each 1 ≤ *i* ≤ *m* + 1 are accepted and $C_{\max}(\bar{R})\geq 2A^{m^{2}+m+1}$.

**Proof**

If there exists some job *J*_2*i*−1_(1 ≤ *i* ≤ *m* + 1) being rejected, then we have $C_{\max}(\bar{R})+\sum_{J_{j}\in R}e_{j}\geq e_{2i-1}$ = 2*A* ^*m*^2^+*m*+1^ + *A* > *Y*. It is a contradiction. Since job *J*_2*m*+1_ is accepted, we have $C_{\max}(\bar{R})\geq r_{2m+1}(1+b_{2m+1})\geq 2A^{m^{2}+m+1}$.

From Claim 1, we can obtain that only the jobs {*J*_2*i*_ : 1 ≤ *i* ≤ *m*} can be rejected.

**Claim 2 .**

Only job *J*_2*m*+1_ is processed in the last batch.

**Proof**

If there exists a job *J*_*i*_ (1 ≤ *i* ≤ 2*m*) with *J*_2*m*+1_ processed in the same batch *B*, then we have

(2) Cmax⁡=r2m+1(1+b(B))=r2m+1(1+bi)≥Am2+m+1(1+A2−1)=Am2+m+3>Y.

Then, we denote by *B*_*m*+1_ the last batch containing only job *J*_2*m*+1_.

**Claim 3 .**

Each batch contains either a single job *J*_2*i*−1_ or two jobs {*J*_2*i*−1_, *J*_2*i*_} for 1 ≤ *i* ≤ *m*.

**Proof**

For job *J*_2*m*−1_, if there exists a job *J*_*i*_ (1 ≤ *i* ≤ 2*m* − 2) with *J*_2*m*−1_ which are processed in the same batch *B*, then we have *C*_max⁡_ = *r*_2*m*−1_(1 + *b*(*B*)) = *r*_2*m*−1_(1 + *b*_*i*_) ≥ *A* ^*m*^2^+*m*−2^(1 + *A* ^4^ − 1) = *A* ^*m*^2^+*m*+1^ · *A* > *Y*.

Thus, we denote by *B*_*m*_ the new batch containing job *J*_2*m*−1_.

For job *J*_2*m*−3_, if there exists a job *J*_*i*_ (1 ≤ *i* ≤ 2*m* − 4) with *J*_2*m*−3_ which are processed in the same batch *B*_*m*−1_, then we have the completion time of the batch as

(3) C(Bm−1)≥r2m−3(1+b(Bm−1))=r2m−3(1+bi)≥Am2+m−6(1+A6−1)=Am2+m.

The completion time of batch *B*_*m*_ is

(4) C(Bm)=max⁡{C(Bm−1),r2m−1}(1+b(Bm))≥Am2+m+2>Y.

Therefore, batch *B*_*m*−1_ can only contain either job *J*_2*m*−3_ or jobs {*J*_2*m*−3_, *J*_2*m*−4_}. Repeat this deduction, we have the correctness of Claim 3. Let *B*_*i*_ be the batch containing job *J*_2*i*−1_ for 1 ≤ *i* ≤ *m*.

By Claims 2 and 3, we have batch *B*_*i*_ = {*J*_2*i*−1_, *J*_2*i*_} if *J*_2*i*_ is accepted and batch *B*_*i*_ = {*J*_2*i*−1_} if *J*_2*i*_ is rejected. Let *S* = {*i* : *J*_2*i*_ is rejected for 1 ≤ *i* ≤ *m*}. Next, we are ready to show that *S* is a solution of the Equal Products Problem.

Since $C_{\max}(\bar{R})\geq 2A^{m^{2}+m+1}$ and $C_{\max}(\bar{R})+\sum_{J_{j}\in R}e_{j}\leq Y=2A^{m^{2}+m+1}+\ln A$, we have ∑_*j*∈*S*_ln⁡*a*_*j*_ = ∑_*J*_2*j*_∈*R*_ *e*_2*j*_ ≤ ln⁡*A*. If ∏_*j*∈*S*_ *a*_*j*_ = *X* < *A*, we have

(5) Cmax⁡(R¯)+∑Jj∈Rej =max⁡⁡{r(Bm+1),C(Bm)}(1+bm+1)+∑j∈Sln⁡aj =2max⁡⁡{Am2+m+1,Am2+m·∏i∈S¯ai}  +ln⁡X>2Am2+m+1·AX+ln⁡X>Y.  

It is a contradiction. Hence, we have ∏_*j*∈*S*_ *a*_*j*_ = *A* and *S* is a solution of the Equal Products Problem. This completes the proof.

### 3.2. Dynamic Programming Algorithms

**Lemma 3 .**

For problem $1\left|p\text{-}batch,r_{j},p_{j}=b_{j}t,rej,b\geq n\right|C_{\max}(\bar{R})+\sum_{J_{j}\in R}e_{j}$, there exists an optimal schedule such that the accepted jobs are processed in a nonincreasing order of deteriorating rates.

**Proof**

Otherwise, there exist two accepted jobs *J*_*i*_ and *J*_*j*_ such that *J*_*j*_ is processed later than *J*_*i*_, but *b*_*i*_ < *b*_*j*_. Since *b* ≥ *n*, there is no limit on the number of jobs in a batch and we can put job *J*_*i*_ into the batch containing job *J*_*j*_. This does not delay the completion time of any batch. A finite number of repetitions of this procedure yields an optimal schedule of the required form.

Based on Lemma 3, we only consider the schedules in which the accepted jobs are processed in a nonincreasing order of deterioration rates. Assume that jobs are indexed such that *b*_1_ ≥ ⋯≥*b*_*n*_.

Let *F*(*j*, *k*, *E*) denote the optimal objective function value of the following scheduling problem:

- the jobs in consideration are *J*_1_,…, *J*_*j*_;
- *J*_*k*_ is the job with the minimum index in the last batch;
- the total penalty of rejected jobs is *E*.

In any such schedule, there are two possible cases: either *J*_*j*_ is rejected or *J*_*j*_ is accepted and processed on the batching machine. We distinguish two cases in the following discussion.

**Case 1 .**

Job *J*_*j*_ is rejected. Since *J*_*j*_ is rejected, in the corresponding optimal schedule for *J*_1_,…, *J*_*j*−1_, *J*_*k*_ is still the job with the minimum index in the last batch and the total rejection penalty among *J*_1_,…, *J*_*j*−1_ is *E* − *e*_*j*_. Therefore, we have *F*(*j*, *k*, *E*) = *F*(*j* − 1, *k*, *E* − *e*_*j*_) + *e*_*j*_.

**Case 2 .**

Job *J*_*j*_ is accepted. In this case, we consider this problem in the following two cases.

*Case 2* *.1*. If *k* = *j*, then job *J*_*j*_ starts a new batch. The starting time of job *J*_*j*_ is max⁡{*F*(*j* − 1, *k*′, *E*) − *E*, *r*_*j*_}. Therefore, we have *F*(*j*, *k*, *E*) = min⁡_0≤*k*′≤*j*−1_⁡{max⁡{*F*(*j* − 1, *k*′, *E*) − *E*, *r*_*j*_}(1 + *b*_*j*_) + *E*}.

*Case 2.2*. If *k* < *j*, then job *J*_*j*_ can be assigned to the last batch containing job *J*_*k*_. Therefore, *F*(*j*, *k*, *E*) = max⁡{(*F*(*j* − 1, *k*, *E*) − *E*)/(1 + *b*_*k*_), *r*_*j*_}(1 + *b*_*k*_) + *E*.

Combining the above two cases, we design the following dynamic programming algorithm.

*Algorithm DP1*. Consider the following steps.

*Step 1 (initialization)*. Consider *F*(1,1, 0) = *r*_1_(1 + *b*_1_) and *F*(1, *k*, *E*) = *∞* for (*k*, *E*)≠(1,0).

Consider *F*(1,0, *e*_1_) = *t*_0_ + *e*_1_ and *F*(1, *k*, *E*) = *∞* for (*k*, *E*)≠(0, *e*_1_).

*Step 2 (recursion)*. If *k* = *j*,

(6) F(j,k,E) =min⁡{min⁡0≤k′≤j−1{max⁡{F(j−1,k′,E)−E,rj}           ×(1+bj)+E},F(j−1,k,E−ej)+ej}.

If *k* < *j*,

(7) F(j,k,E)=min⁡{max⁡{F(j−1,k,E)−E1+bk,rj}(1+bk)     +E,F(j−1,k,E−ej)+ej}.

*Step 3 (optimal solution)*. The optimal value is given by min⁡{*F*(*n*, *k*, *E*) : 0 ≤ *k* ≤ *n*, 0 ≤ *E* ≤ ∑_*j*=1_^*n*^ *e*_*j*_}.

**Theorem 4 .**

Algorithm* DP1* solves the problem $1\left|p\text{-}batch,r_{j},p_{j}=b_{j}t,rej,b\geq n\right|C_{\max}(\bar{R})+\sum_{J_{j}\in R}e_{j}$ in *O*(*n* ^2^∑_*j*=1_^*n*^ *e*_*j*_) time.

**Proof**

The correctness of Algorithm DP1 is guaranteed by the above discussion. The recursive function has at most *O*(*n* ^2^∑_*j*=1_^*n*^ *e*_*j*_) states; each iteration takes a constant time to execute. Hence, the running time of Algorithm DP1 is *O*(*n* ^2^∑_*j*=1_^*n*^ *e*_*j*_).

Next, we give another dynamic programming Algorithm DP2. Let *f*(*j*, *k*, *E*) denote the minimum value of the maximum completion time satisfying the following conditions.

- The jobs in consideration are *J*_1_,…, *J*_*j*_.
- *J*_*k*_ is the job with the minimum index in the last batch.
- The total penalty of rejected jobs is *E*.

In any such schedule, there are two possible cases: either *J*_*j*_ is rejected or *J*_*j*_ is accepted and processed on the batching machine.

**Case 1 .**

If job *J*_*j*_ is rejected, we have *f*(*j*, *k*, *E*) = *f*(*j* − 1, *k*, *E* − *e*_*j*_).

**Case 2 .**

Job *J*_*j*_ is accepted.

*Case 2.1*. If *k* = *j*, then job *J*_*j*_ starts a new batch; the starting time of job *J*_*j*_ is max⁡{*f*(*j* − 1, *k*′, *E*), *r*_*j*_}. Therefore, we have *f*(*j*, *k*, *E*) = min⁡_0≤*k*′≤*j*−1_⁡{max⁡{*f*(*j* − 1, *k*′, *E*), *r*_*j*_}(1 + *b*_*j*_)}.

*Case 2.2*. If *k* < *j*, then job *J*_*j*_ can be assigned to the last batch containing job *J*_*k*_. Therefore, *f*(*j*, *k*, *E*) = max⁡{*f*(*j* − 1, *k*, *E*)/(1 + *b*_*k*_), *r*_*j*_}(1 + *b*_*k*_).

Combining the above two cases, we design the dynamic programming algorithm as follows.

*Algorithm DP2*. Consider the following steps.

*Step 1 (initialization)*. Consider *f*(1,1, 0) = *r*_1_(1 + *b*_1_) and *f*(1, *k*, *E*) = *∞* for any (*k*, *E*)≠(1,0).

Consider *f*(1,0, *e*_1_) = *t*_0_ and *f*(1, *k*, *E*) = *∞* for any (*k*, *E*)≠(0, *e*_1_).

*Step 2 (recursion)*. If *k* = *j*,

(8) f(j,k,E) =min⁡{min⁡0≤k′≤j−1{max⁡{f(j−1,k′,E),rj}(1+bj)},     f(j−1,k,E−ej)}.

If *k* < *j*,

(9) f(j,k,E) =min⁡⁡{max⁡⁡{f(j−1,k,E)1+bk,rj}(1+bk),      f(j−1,k,E−ej)}.

*Step 3 (optimal solution)*. The optimal value is determined by min⁡{*f*(*n*, *k*, *E*) + *E* : 0 ≤ *k* ≤ *n*, 0 ≤ *E* ≤ ∑_*j*=1_^*n*^ *e*_*j*_}.

**Theorem 5 .**

Algorithm DP2 solves the problem $1|p\text{-}batch,r_{j},p_{j}=b_{j}t,rej,b\geq n|C_{\max}(\bar{R})+\sum_{J_{j}\in R}e_{j}$ in *O*(*n* ^2^∑_*j*=1_^*n*^ *e*_*j*_) time.

### 3.3. An FPTAS

Algorithm *A* is a *ρ*-approximation (*ρ* ≥ 1) algorithm for a minimization problem if it produces a solution that is at most *ρ* times than the optimal one (*ρ* is also referred to as the worst-case ratio). A family of algorithms {*A*_*ϵ*_ : *ϵ* > 0} is called a fully polynomial-time approximation scheme (FPTAS), if, for each *ϵ* > 0, the algorithm *A*_*ϵ*_ is a (1 + *ϵ*)-approximation algorithm running in polynomial time in the input size and 1/*ϵ*. In the sequel, we assume 0 < *ϵ* ≤ 1.

For any *ϵ*′ > 0 and *x* ≥ 1, if (1 + *ϵ*′)^*k*−1^ < *x* < (1 + *ϵ*′)^*k*^, then we define ⌈*x*⌉_*ϵ*′_ = (1 + *ϵ*′)^*k*^, ⌊*x*⌋_*ϵ*′_ = (1 + *ϵ*′)^*k*−1^. If *x* is an exact power of (1 + *ϵ*′), then ⌈*x*⌉_*ϵ*′_ = ⌊*x*⌋_*ϵ*′_ = *x*. Note that ⌈*x*⌉_*ϵ*′_ ≤ (1 + *ϵ*′)*x* for any *x* ≥ 1.

For any *ϵ* > 0, let *ϵ*′ = *ϵ*/2*n* and let *R* = {*J*_*i*_1__, *J*_*i*_2__,…, *J*_*i*_*m*__} be the set of the rejected jobs, where *i*_1_ < *i*_2_ < ⋯<*i*_*m*_. The inflated rejection penalty of the set *R* with respect to any *ϵ*′ > 0 is defined as

(10) $$\begin{array}{c} \phi_{\epsilon^{\prime }}\left(R\right)=\{\begin{array}{cc} {\left\lceil e_{i_{m}}+\phi_{\epsilon^{\prime }}(R-J_{i_{m}})\right\rceil }_{\epsilon^{\prime }}, & \text{if}\;\;m\geq 1,\\ 0, & \text{if}\;\;R=\emptyset . \end{array} \end{array}$$

**Lemma 6 (see [25]).**

For any set *R* of jobs and any *ϵ*′ > 0, *ϕ*_*ϵ*′_(*R*)≤(1 + *ϵ*′)^|*R*|^∑_*J*_*j*_∈*R*_ *e*_*j*_.

**Lemma 7 (see [19]).**

For any 0 < *x* ≤ 1 and for any integer *n* ≥ 1, (1 + (*x*/*n*))^*n*^ ≤ 1 + 2*x* holds.

Based on Lemmas 6 and 7, we have the following theorem.

**Theorem 8 .**

For any 0 < *ϵ* ≤ 2, the optimal objective function value for $1\left|p\text{-}batch,r_{j},p_{j}=b_{j}t,rej,b\geq n\right|C_{\max}(\bar{R})+\phi_{\epsilon^{\prime }}(R)$ is at most (1 + *ϵ*) times the optimal objective function value for $1\left|p\text{-}batch,r_{j},p_{j}=b_{j}t,rej,b\geq n\right|C_{\max}(\bar{R})+\sum_{J_{j}\in R}e_{j}$.

Assume that the jobs have been indexed such that *b*_1_ ≥ ⋯≥*b*_*n*_ and define

(11) $$\begin{array}{c} \tau_{l}=\{\begin{array}{cc} {\left(1+\epsilon^{\prime }\right)}^{l}, & \text{if}\;\;l\neq -1,\\ 0, & \text{if}\;\;l=-1. \end{array} \end{array}$$

Let *f*(*j*, *k*, *l*) denote the minimum value of the maximum completion time satisfying the following conditions.

- The jobs in consideration are *J*_1_,…, *J*_*j*_.
- In the last batch, *J*_*k*_ is the job with the minimum index.
- The inflated rejection penalty of the rejected jobs is *τ*_*l*_.

We design a dynamic programming algorithm as follows.

*Algorithm A*_*ϵ*_. Consider the following steps.

*Step 1 (initialization)*. Consider

(12) $$\begin{array}{c} f\left(1,k,l\right)=\{\begin{array}{cc} t_{0}, & \text{if}\;\;\tau_{1}={\left\lceil e_{1}\right\rceil }_{\epsilon^{\prime }},\\ r_{1}\left(1+b_{1}\right), & \text{if}\;\;l=-1,\\ +\infty , & \text{otherwise}. \end{array} \end{array}$$

*Step 2 (recursion)*. If *k* = *j*, then

(13) f(j,k,l) =min⁡{min⁡0≤k′≤j−1{max⁡{f(j−1,k′,l),rj}(1+bj)},     min⁡−1≤l′≤l~f(j−1,k,l′)}.

If *k* < *j*, then

(14) f(j,k,l)=min⁡{max⁡{f(j−1,k,l)1+bk,rj}(1+bk),     min⁡−1≤l′≤l~f(j−1,k,l′)},

where $\tilde{l}$ is given by ${\left(1+\epsilon^{\prime }\right)}^{\tilde{l}}={\left\lfloor {\left(1+\epsilon^{\prime }\right)}^{l}-e_{j}\right\rfloor }_{\epsilon^{\prime }}$.

*Step 3 (output)*. The optimal value is determined by min⁡{*f*(*n*, *k*, *l*) + *τ*_*l*_ : 0 ≤ *k* ≤ *n*, −1 ≤ *l* ≤ *L*}, where *L* is the smallest integer such that (1 + *ϵ*′)^*L*^ ≥ (1 + *ϵ*′)^*n*^∑_*j*=1_^*n*^ *e*_*j*_.

**Theorem 9 .**

There exists an FPTAS for problem $1|p\text{-}batch,r_{j},p_{j}=b_{j}t,rej,b\geq n|C_{\max}(\bar{R})+\sum_{j\in R}e_{j}$ which runs in *O*((*n* ^4^/*ϵ* ^2^)log⁡^2^∑_*j*=1_^*n*^ *e*_*j*_) time.

**Proof**

The correctness of Algorithm *A*_*ϵ*_ is guaranteed by the above discussion. We need to compute exactly *O*(*n* ^2^(*L* + 2)) values *f*(*j*, *k*, *l*) and computation of each value takes *O*(*L*) time, where *L* = *O*((*n*/*ϵ*)log⁡∑_*j*=1_^*n*^ *e*_*j*_). Hence, the running time of Algorithm *A*_*ϵ*_ is *O*(*n* ^2^ *L* ^2^) = *O*((*n* ^4^/*ϵ* ^2^)log⁡^2^∑_*j*=1_^*n*^ *e*_*j*_).

### 3.4. An Optimal Algorithm of One Special Case

The special case where all jobs have the same release dates is considered in this subsection, that is, the problem $1\left|p\text{-batch},r_{j}=t_{0},p_{j}=b_{j}t,rej,b\geq n\right|C_{\max}(\bar{R})+\sum_{J_{j}\in R}e_{j}$.

We first reindex the jobs in nonincreasing order of their deteriorating rates such that *b*_1_ ≥ ⋯≥*b*_*n*_.

**Lemma 10 .**

For problem $1\left|p\text{-}batch,r_{j}=t_{0},p_{j}=b_{j}t,rej,b\geq n\right|C_{\max}(\bar{R})+\sum_{J_{j}\in R}e_{j}$, in any optimal schedule, if job *J*_*j*_  (1 ≤ *j* ≤ *n*) is accepted, then the jobs set {*J*_*i*_ : *i* ≥ *j*} is accepted too.

**Proof**

If job *J*_*j*_ is accepted, since *b* ≥ *n*, there is no limit on the number of jobs in a batch; the jobs {*J*_*j*+1_,…, *J*_*n*_} can be accepted and assigned to the current last batch. Obviously, this does not increase the value of the objective function. This completes the proof.

**Theorem 11 .**

For problem $1\left|p\text{-}batch,r_{j}=t_{0},p_{j}=b_{j}t,rej,b\geq n\right|C_{\max}(\bar{R})+\sum_{J_{j}\in R}e_{j}$, the optimal function value is min⁡{*t*_0_(1 + *b*_1_), ∑_*j*=1_^*n*^ *e*_*j*_, min⁡_2≤*j*≤*n*_⁡{*t*_0_(1 + *b*_*j*_) + ∑_*i*=1_^*j*−1^ *e*_*i*_}}; the optimal solution can be found in *O*(*n*log⁡*n*) time.

## 4. Conclusion

In this paper, we focus on the unbounded parallel batch scheduling of deteriorating jobs with release date and rejection. The objective is to minimize the makespan of the accepted jobs plus the total penalty of rejected jobs. We show that it is binary NP-hard; we provide two pseudopolynomial time algorithms and also propose a fully polynomial-time approximation scheme for the problem $1\left|p\text{-batch},r_{j},p_{j}=b_{j}t,rej,b\geq n\right|C_{\max}(\bar{R})+\sum_{J_{j}\in R}e_{j}$. Furthermore, we show that this problem can be solvable in *O*(*n*log⁡*n*) time when jobs have identical release dates. For future research, it would be interesting to focus on scheduling deteriorating jobs with other objectives. Analysis of the other models of scheduling with rejection is also an interesting research direction.
